# Impact of Emergency Department Crowding on Discharged Patient Experience

**DOI:** 10.5811/westjem.2022.10.58045

**Published:** 2022-12-31

**Authors:** Yosef Berlyand, Martin S. Copenhaver, Benjamin A. White, Sayon Dutta, Joshua J. Baugh, Susan R. Wilcox, Brian J. Yun, Ali S. Raja, Jonathan D. Sonis

**Affiliations:** * Massachusetts General Hospital, Department of Emergency Medicine, Boston, Massachusetts; † Harvard Medical School, Boston, Massachusetts; ‡ Healthcare Systems Engineering, Massachusetts General Hospital, Boston, Massachusetts; § Boston Medical Center, Department of Emergency Medicine, Boston, Massachusetts

## Abstract

**Introduction:**

While emergency department (ED) crowding has deleterious effects on patient care outcomes and operational efficiency, impacts on the experience for patients discharged from the ED are unknown. We aimed to study how patient-reported experience is affected by ED crowding to characterize which factors most impact discharged patient experience.

**Methods:**

This institutional review board-exempt, retrospective, cohort study included all discharged adult ED patients July 1, 2020–June 30, 2021 with at least some response data to the the National Research Corporation Health survey, sent to most patients discharged from our large, academic medical center ED. Our query yielded 9,401 unique encounters for 9,221 patients. Based on responses to the summary question of whether the patient was likely to recommend our ED, patients were categorized as “detractors” (scores 0–6) or “non-detractors” (scores 7–10). We assessed the relationship between census and patient experience by 1) computing percentage of detractors within each care area and assessing for differences in census and boarder burden between detractors and non-detractors, and 2) multivariable logistic regression assessing the relationship between likelihood of being a detractor in terms of the ED census and the patient’s last ED care area. A second logistic regression controlled for additional patient- and encounter-specific covariates.

**Results:**

Survey response rate was 24.8%. Overall, 13.9% of responders were detractors. There was a significant difference in the average overall ED census for detractors (average 3.70 more patients physically present at the time of arrival, 95% CI 2.33–5.07). In unadjusted multivariable analyses, three lower acuity ED care areas showed statistically significant differences of detractor likelihood with changes in patient census. The overall area under the curve (AUC) for the unadjusted model was 0.594 (CI 0.577–0.610). The adjusted model had higher AUC (0.673, CI 0.657–.690]; P<0.001), with the same three care areas having significant differences in detractor likelihood based on patient census changes. Length of stay (OR 1.71, CI 1.50–1.95), leaving against medical advice/without being seen (OR 5.15, CI 3.84–6.89), and the number of ED care areas a patient visited (OR 1.16, CI 1.01–1.33) was associated with an increase in detractor likelihood.

**Conclusion:**

Patients arriving to a crowded ED and ultimately discharged are more likely to have negative patient experience. Future studies should characterize which variables most impact patient experience of discharged ED patients.

## INTRODUCTION

Emergency department (ED) crowding continues to be a major challenge in the United States, with important ramifications for patient experience, care quality, and staff experience.^1^–^9^ Crowding has been shown to have deleterious effects on patient care outcomes and operational efficiency.^4^,^6^,^8^–^17^ There have been numerous efforts to mitigate ED crowding such as leveraging alternative pathways to avoid hospital admissions, creation of full-capacity protocols to increase inpatient availability of beds, opening of nearby urgent care centers to offload low-acuity volume, and protocols triggering reductions in outside hospital transfers, direct admissions, and elective procedures.^18^–^28^

While ED crowding has multiple negative operational impacts, the impact on patient experience for ED patients who are ultimately discharged has not been well studied. While long waits and throughput times have been shown to negatively impact experience, the aspects of crowding that most directly impact the experience of discharged ED patients are poorly understood. Several methods for modeling ED crowding have been previously used including index functions taking into account multiple variables,^11^,^14^,^29^–^31^ and simple measures such as the ED occupancy rate,^32^ boarder burden in the ED,^8^ or the number of concurrent ED arrivals, but none have been shown to impact patient experience.^16^,^33^

A boarding inpatient in the ED (“boarder”) is frequently defined as a patient who remains in the ED more than two hours after an inpatient bed request has been placed.^8^ Boarding inpatients occupy space and use scarce resources including nursing and clinician bandwidth that would otherwise be used for evaluation of new ED patients. A prior study from our ED found that increased inpatient boarders resulted in an increased length of stay (LOS) for patients who were discharged from the ED, demonstrating a negative impact of boarding on even low-acuity patients.^15^

It is not known whether the operational impacts of crowding result in a worsened patient experience for patients discharged from the ED. We aimed to study how patient-reported experience is affected by ED crowding as measured by the ED census and boarder burden to better characterize which factors most impact discharged patient experience. We hypothesized that worsened ED crowding negatively impacts patient experience for patients discharged from the ED.

## METHODS

This study was evaluated by our Institutional Human Research Committee and deemed exempt from institutional review board review.

### Setting

This study was conducted at a large academic medical center which is a Level I adult and pediatric trauma center, STEMI-receiving center, and stroke center with approximately 110,000 annual ED visits and 1,019 licensed operational beds. Patients in our ED are triaged by acuity into several care areas (Table 1).

**Population Health Research Capsule**

What do we already know about this issue?

*Emergency department (ED) crowding has been shown to negatively impact patient care outcomes and operational efficiency for admitted patients*.

What was the research question?

*We sought to establish whether crowding results in a worsened experience for patients discharged from the ED*.

What was the major finding of the study?

*Discharged patients are more likely to be identified as detractors if crowding is worse, with an average greater census at the time of their arrival by 3.70 patients (95% CI 2.33–5.07)*.

How does this improve population health?

*Characterizing how ED crowding impacts discharged patient experience is vital to ensure that operational interventions are impactful in improving patient experience*.

### Data Collection

Most patients discharged from our ED are subsequently sent an electronic survey to assess their patient experience, which is produced, managed, and administered by the third-party organization National Research Corporation (NRC) Health (Lincoln, NE). The survey is sent to all adult patients within three days of their encounter, unless they meet the following exclusion criteria: surveyed for another encounter within the prior three days or the same department or clinician within the prior six months, previously requested to be excluded from a NRC Health survey, confidential patients (including well known individuals or prisoners), or absent contact information.

The NRC Health survey is administered in Arabic, Chinese (Mandarin), Russian, Portuguese, Haitian Creole, Spanish, Khmer, and English according to the patient’s listed preferred language in the electronic health record (EHR). If a language listed is not one of those eight languages, then the survey is administered in English. The surveys are administered by email or interactive voice response (IVR) by phone with the exception of Arabic, which is only administered by IVR. Patients must complete the survey within 15 days of receipt.

This survey includes both quantitative data and qualitative comments. Quantitative data is summarized by a variable called the “net promotor score,” which is generated from the patient’s response to the summary question of whether they are likely to recommend our ED on a scale of 0–10. Scores of 0–6 are categorized as “detractors,” scores of 7–8 are “passive,” and scores of 9–10 are “promotors.” Among patients with at least some survey response data, we defined the responses as binary for “detractors” (ie, scores of 0–6) and “non-detractors.” Non-detractors also included non-respondents for the specific recommendation question.

**Figure 1:**
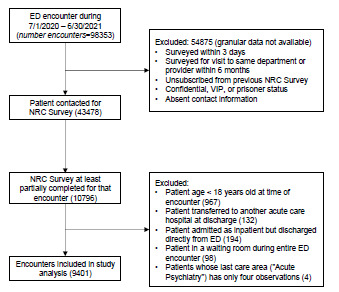
Inclusion criteria flow chart. *ED*, emergency department, *NRC*, National Research Corporation.

We queried NRC Health survey data to find all ED encounters with available NRC data from July 1, 2020 – June 30, 2021 and for which both a) the patient was at least 18 years old at the time of the ED encounter, and b) the patient was discharged directly from the ED without being admitted as an inpatient (ie, the patient was not admitted to the hospital; transferred from the ED to a procedural area; admitted as an inpatient while in the ED but directly discharged home from the ED, or transferred to another acute care hospital), yielding a total of 9,401 unique encounters for 9,221 patients (Figure 1). Using encounter-specific identifiers, we linked each survey response to the patient’s EHR (using an internal data warehouse) to obtain pertinent patient demographics, encounter-specific data such as ED LOS, and operational variables at the time of the patient’s arrival to their final care location in the ED, including ED census and the number of ED boarders. The ED census and ED boarder burden were measured within the specific location that was the patient’s last care area prior to discharge. Boarders were defined as inpatients with a bed request in place for greater than two hours who remained in the ED. These operational metrics were computed based on the census of all patients in the ED.

### Statistical Analysis

To assess the relationship between census and patient experience, we conducted descriptive and predictive statistical analyses. For the descriptive analysis, we computed the percentage of detractors among the survey respondents as well as summary data on the associated ED census and ED boarder census at the time of each patient’s arrival to their final care area (including differences between detractors and non-detractors). We also performed a multivariable logistic regression analysis to assess the relationship between a patient’s likelihood of being a detractor (as an outcome) in terms of the ED census and the patient’s care area in the ED. In addition to this model, we also estimated a second logistic regression model that controls for a variety of additional patient- and encounter-specific covariates, including the number of distinct ED care areas and waiting rooms the patient visited during their encounter, their age, their gender, whether their NRC Health survey was conducted in English, whether the patient was placed in observation status or in a hallway bed during their encounter, whether the patient left against medical advice (AMA) or without being seen by a clinician, and finally the (logarithm of) LOS in hours. To enable comparison across ED areas, all area-specific censuses were standardized (ie, the mean for that area was subtracted, and the result then divided by that area census’ standard deviation).

We evaluated the discriminative performance of the predictive models using the area under the receiver operating characteristic curve (AUC), also known as the C-statistic or concordance statistic, a standard measure for assessing the ability of classification models to identify a binary outcome. Coefficients of the models are presented in terms of odds ratios (for detractors relative to non-detractors), and all confidence intervals (CI) were reported at the 95% level. We conducted all statistical analyses in R version 4.1.3 (R Foundation for Statistical Computing, Vienna, Austria).^34^ In the Supplement we also include several other logistic regression models, which distinguish between boarder and non-boarder patient census.

## RESULTS

### Descriptive Analysis

For the period studied, the survey response rate was 24.8%. A summary of detractor characteristics and differences in patient census and boarder-specific census is shown in Table 2. Overall, 13.9% of survey responders were detractors, with significant variability across the different ED locations (lowest in Care Area G and highest in Care Area B). Further, there was a significant difference in the average overall ED census for detractors (an average of 3.70 more patients, (95% CI 2.33–5.07), with the relative magnitude of the effect varying by care area. There was significant variability in terms of the boarder census across locations (such as Care Area F, with a large proportion of boarders, vs Care Area D).

### Predictive Analysis

**Figure 2:**
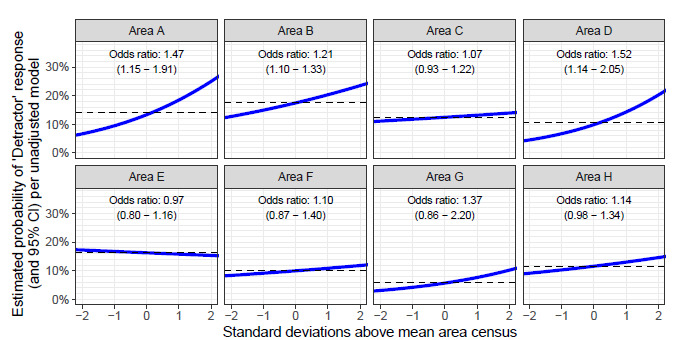
Estimated probability of detractor survey response as predicted by care area census. *CI*, confidence interval

The coefficients of the two multivariable logistic regression models are shown in Table 3. The AUC for the unadjusted model, based on each patient’s last ED location and the census of that area at the patient’s arrival, was 0.594 (CI 0.577–0.610). Three locations showed statistically significant differences in the odds ratios of detractor likelihood with changes in the area’s patient census: Care Area A (OR 1.47, CI 1.15–1.91), Care Area B (OR 1.21, CI 1.10–1.33), and Care Area D (OR 1.52, CI 1.14–2.05) (Table 3 and Figure 2). In contrast, the adjusted model (which controls for several patient- and encounter-specific covariates) has a higher AUC compared with our unadjusted model 0.673 [0.657–0.690], *P*<0.001, *cf*. Supplement, Supporting Table 2), with the same three locations having a significant difference for changes in patient census: Care Area A (1.34 [1.04–1.74]), Care Area B (1.15 [1.04–1.27]), and Care Area D (1.38 [1.03–1.87]).

Among encounter-related covariates in the adjusted model, three were significant: LOS (1.71 [1.50–1.95], Table 3); leaving AMA or leaving without being seen (LWBS) (5.15 [3.84–6.89], Table 3); and the number of distinct ED care areas a patient visits (1.16 [1.01–1.33], Table 3). Several other measures (number of distinct waiting rooms a patient visits, whether patient is placed in a hallway bed, and whether patient is placed in observation status during their encounter) were not. The three patient-specific covariates were all significant in the adjusted model (age, gender, and whether the patient’s survey was conducted in English).

## DISCUSSION

In this retrospective cohort study, we aimed to assess how the patient-reported experience of discharged ED patients is impacted by ED crowding as measured by ED census and boarder burden. Overall, we found that discharged patients are more likely to have a negative patient experience if ED crowding is worse at the time of their arrival. We found that within our lower acuity care areas (Care Area A, Care Area B, and Care Area D) increased ED census at the time of the patient’s arrival increased the likelihood of the patient being a detractor as measured by the net promotor score. Moreover, discharged patient experience was generally rated lower in the lower acuity care areas as compared to the higher acuity care areas. There was no statistically significant impact of patient census on patient experience in the higher acuity care areas.

Given the myriad known effects of ED crowding on operational metrics and clinical outcomes, it is unsurprising that discharged ED patients feel the impact of crowding and have a worsened patient experience when ED resources are stretched thin. Our findings suggest that although ED crowding increases the likelihood of a patient reporting a negative experience, there are many variables that impact patient experience that we are not capturing in our surveys and data. Our fully adjusted model considered several potential confounding factors, such as age, patient gender, and whether a patient ultimately left AMA or without being seen by a clinician. The fully adjusted model did show an increased AUC compared with our unadjusted model, concomitant with a decrease in the odds ratios for the three areas with significant differences.

This attenuation in odds ratios is expected given the partially mediating influence of several of the covariates included in the adjusted model. For example, increased LOS is well known to be correlated with increased measures of ED crowding, 35 and we found that increase as well. Likewise, we also saw that a patient leaving AMA or LWBS has a large-magnitude odds ratio for being a detractor in our model, and increased AMA/LWBS rates are associated with crowding as well.^1^ Despite including these covariates, the AUC for our model was 0.673 [0.657–0.690], suggesting that a large portion of the variation in a patient being a detractor is unexplained by our model. We suspect that some of this variation would be explained by other confounding variables that we were unable to measure, such as time until imaging acquisition or time until completion of specialty consultation. Other potential variables, which may explain some of this variation, may be more difficult to measure with our existing surveys, such as the way in which clinician experience on crowded days manifests itself in patient interactions.

## LIMITATIONS

This study had several limitations. The primary limitation was that a large majority of discharged patients (86.1%) were non-detractors, limiting our ability to assess factors that predict being a detractor. While statistically significant, the effect size of the ED census on patient experience was rather small. There are also standard limitations associated with using survey data, as patient populations with limited access to technology or with unstable housing are less likely to respond to the survey. Finally, this was a retrospective, single-site study, which limits the generalizability of our results.

Although ED crowding has previously been clearly associated with several negative clinical and operational outcomes, as well as worsened patient experience for admitted patients,^36^ this is the first study we are aware of that specifically illustrates the impact of ED crowding on the experience of discharged patients. As most patients seen in the ED are ultimately discharged, and discharged patients represent the unique group whose experience is limited to their time in the ED, their experience should be of particular interest to ED leaders seeking to measure the impact of interventions or improvement efforts. Intuitively, we thought it was likely that ED crowding would indeed lead to worsened patient experience. We were surprised, however, by the degree of variance in the data, even with adjustment for covariates commonly thought to impact patient experience.

## CONCLUSION

Our study shows that patients who arrive to a crowded ED and are ultimately discharged are more likely to have a negative patient experience than those who arrive at times of less crowding. It is, therefore, important that we continue to combat ED crowding and boarding to improve discharged patient experience. Future studies are needed to understand whether our results are generalizable to other ED settings, to identify underlying sources of variation in patient experience based on care area characteristics, and to better characterize which variables are most impactful on the patient experience of discharged ED patients.

## Supplementary Information



## Figures and Tables

**Table 1:** Description of emergency department care areas.

Care area	Brief description
A	Care area for rapid clinician assessment and intervention for patients not requiring higher acuity resources.
B	Care area for continuation of care for patients initially evaluated in Care Area A who are able to sit in this internal waiting space while awaiting testing results and/or consultation. There are a limited number of curtained bed spaces that can be used to care for non-ambulatory patients or boarding inpatients.
C	Care area for patients with a single-system complaint and without need for continuous monitoring.
D	Care area for low-acuity, ambulatory patients with single-system complaints that do not require monitoring. Primary focuses include stable orthopedic evaluations and minor procedures such as laceration repairs and abscess drainages.
E	Care area for Intermediate acuity patients with cardiopulmonary monitoring capabilities. Patients are not hemodynamically unstable and do not require immediate resuscitation capabilities.
F	Care area for major resuscitation of the highest acuity patients.
G	Care area for patients under the age of 22 who are behaviorally appropriate and do not require the resuscitation capabilities of Care Area F.
H	Emergency department observation unit designed for the continuation of care for patients with an expected length of stay less than 48 hours.

**Table 2:** Summary statistics on survey response and average number of patients (and boarders) at the time of a patient’s arrival to their last care area.*

Last care area	Number of encounters with survey data (percent of total)	Detractor percentage	Average number of patients in area (SD)	Difference in area patient census means (SE)	Average number of boarders in area (SD)	Difference in area boarder means (SE)
A	645 (6.86)	13.95	6.20 (2.14)	0.74 (0.22)	0.00 (0.00)	0.00 (0.00)
B	2,907 (30.92)	17.65	26.01 (6.56)	1.22 (0.32)	5.69 (3.29)	0.20 (0.16)
C	1,877 (19.97)	12.47	7.66 (3.18)	0.21 (0.22)	0.31 (0.65)	0.00 (0.04)
D	485 (5.16)	10.52	6.69 (2.96)	1.24 (0.45)	0.01 (0.10)	0.01 (0.02)
E	829 (8.82)	16.28	28.46 (4.54)	−0.16 (0.41)	4.98 (3.65)	−0.66 (0.32)
F	750 (7.98)	10.00	21.31 (5.90)	0.57 (0.73)	5.97 (3.62)	−0.34 (0.39)
G	321 (3.41)	5.92	7.29 (2.72)	0.87 (0.59)	0.35 (0.66)	0.19 (0.24)
H	1,587 (16.88)	11.66	20.77 (4.94)	0.65 (0.40)	1.09 (1.22)	−0.07 (0.09)
*Overall*	9,401 (100.00)	13.85	131.28 (23.71)	3.70 (0.70)	18.77 (10.08)	0.69 (0.31)

* “Detractor percentage” is the percent of detractors among all patients with at least some survey data (i.e., non-response to the facility recommendation question is counted as a non-detractor). Differences are measured as mean patient census in care area for Detractors minus non-detractors. The ‘Overall’ row indicates the number of patients (and boarders, respectively) in the ED in total (ie, not localized to that specific area) at the time of the patient’s arrival to their last care area.

**Table 3:** Logistic regression models for estimating a patient’s detractor likelihood.*

Predictors	Unadjusted model			Adjusted model		
	
	Odds ratios (SE)	CI	*P* value	Odds ratios (SE)	CI	*P* value
(Intercept)	0.15 (0.02)	0.12 – 0.19	<0.001	0.11 (0.02)	0.07 – 0.17	<0.001
Last location						
A	1.00	reference		1.00	reference	
B	1.38 (0.18)	1.07 – 1.78	**0.013**	0.71 (0.11)	0.52 – 0.96	**0.025**
C	0.92 (0.13)	0.71 – 1.22	0.562	0.63 (0.09)	0.47 – 0.85	**0.002**
D	0.71 (0.14)	0.48 – 1.04	0.085	0.63 (0.13)	0.42 – 0.93	**0.023**
E	1.26 (0.19)	0.94 – 1.71	0.125	0.68 (0.12)	0.48 – 0.96	**0.03**
F	0.72 (0.12)	0.51 – 1.00	0.053	0.43 (0.08)	0.30 – 0.63	**<0.001**
G	0.39 (0.11)	0.22 – 0.65	**0.001**	0.19 (0.05)	0.10 – 0.32	**<0.001**
H	0.85 (0.12)	0.65 – 1.13	0.258	0.40 (0.09)	0.26 – 0.62	**<0.001**
Patients in area (standardized) * Last location						
A	1.47 (0.19)	1.15 – 1.91	**0.003**	1.34 (0.17)	1.04 – 1.74	**0.027**
B	1.21 (0.06)	1.10 – 1.33	**<0.001**	1.15 (0.06)	1.04 – 1.27	**0.007**
C	1.07 (0.07)	0.93 – 1.22	0.351	1.09 (0.08)	0.94 – 1.25	0.261
D	1.52 (0.23)	1.14 – 2.05	**0.005**	1.38 (0.21)	1.03 – 1.87	**0.034**
E	0.97 (0.09)	0.80 – 1.16	0.711	0.94 (0.09)	0.78 – 1.13	0.506
F	1.10 (0.13)	0.87 – 1.40	0.427	1.06 (0.13)	0.83 – 1.36	0.622
G	1.37 (0.33)	0.86 – 2.20	0.179	1.33 (0.32)	0.83 – 2.16	0.237
H	1.14 (0.09)	0.98 – 1.34	0.093	1.07 (0.09)	0.91 – 1.25	0.415
Age in years				0.99 (0.00)	0.98 – 0.99	**<0.001**
Gender						
Female				1.00	reference	
Male				0.60 (0.04)	0.52 – 0.67	**<0.001**
Survey completed in English				1.62 (0.16)	1.34 – 1.97	**<0.001**
Number of ED care areas visited				1.16 (0.08)	1.01 – 1.33	**0.031**
Number of ED waiting rooms visited				1.06 (0.07)	0.93 – 1.22	0.38
Placed in Observation status				1.08 (0.15)	0.82 – 1.43	0.575
Placed in hallway bed				1.07 (0.09)	0.91 – 1.25	0.409
Patient leaves AMA or LWBS				5.15 (0.77)	3.84 – 6.89	**<0.001**
Length of stay in hours (logarithm)				1.71 (0.11)	1.50 – 1.95	**<0.001**
AUC (CI)	0.594 (0.577 – 0.610)			0.673 (0.657 – 0.690)		

* The number of patients present in the specific area is standardized (ie, mean is subtracted, and the result is divided by the standard deviation) to allow comparison across different areas; therefore, a unit increase equates to an increase in one standard deviation. P-values below 0.05 are bolded. Odds ratios greater than 1.0 correspond to increased likelihood of being a detractor.

*SE*, standard error; *CI*, confidence interval; *ED*, emergency department; *AMA*, against medical advice; *LWBS*, left without being seen; *AUC*, area under the receiver-operator characteristic curve.

## References

[1] Bernstein SL, Aronsky D, Duseja R (2009). The effect of emergency department crowding on clinically oriented outcomes. Acad Emerg Med.

[2] Miró O, Antonio MT, Jiménez S (1999). Decreased health care quality associated with emergency department overcrowding. Eur J Emerg Med.

[3] Institute of Medicine (2007). Hospital-Based Emergency Care: At the Breaking Point.

[4] Sikka R, Mehta S, Kaucky C (2010). ED crowding is associated with an increased time to pneumonia treatment. Am J Emerg Med.

[5] Chatterjee P, Cucchiara BL, Lazarciuc N (2011). Emergency department crowding and time to care in patients with acute stroke. Stroke.

[6] McCarthy ML, Zeger SL, Ding R (2009). Crowding delays treatment and lengthens emergency department length of stay, even among high-acuity patients. Ann Emerg Med.

[7] Fee C, Weber E, Maak C (2007). Effect of emergency department crowding on time to antibiotics in patients admitted with community-acquired pneumonia. Ann Emerg Med.

[8] White BA, Biddinger PD, Chang Y (2013). Boarding inpatients in the emergency department increases discharged patient length of stay. J Emerg Med.

[9] White BA, Dorner SC, Yun BJ (2018). Quantifying the operational impact of boarding inpatients on emergency department radiology services. Am J Emerg Med.

[10] Jones P, Wells S, Ameratunga S (2018). Towards a best measure of emergency department crowding: lessons from current Australasian practice. Emerg Med Australas.

[11] Jones SS, Allen TL, Flottemesch TJ (2006). An independent evaluation of four quantitative emergency department crowding scales. Acad Emerg Med.

[12] Noel G, Drigues C, Viudes G (2018). Which indicators to include in a crowding scale in an emergency department? A national French Delphi study. Eur J Emerg Med.

[13] Ilhan B, Kunt MM, Damarsoy FF (2020). NEDOCS: Is it really useful for detecting emergency department overcrowding today?. Medicine (Baltimore).

[14] Bernstein SL, Verghese V, Leung W (2003). Development and validation of a new index to measure emergency department crowding. Acad Emerg Med.

[15] CDC National Hospital Ambulatory Medical Care Survey: 2014 emergency department summary tables. Natl Health Stat Report.

[16] Hwang U, McCarthy ML, Aronsky D (2011). Measures of crowding in the emergency department: A systematic review. Acad Emerg Med.

[17] Richardson DB (2006). Increase in patient mortality at 10 days associated with emergency department overcrowding. Med J Aust.

[18] Varney J, Weiland TJ, Jelinek G (2014). Efficacy of hospital in the home services providing care for patients admitted from emergency departments: An integrative review. Int J Evid Based Healthc.

[19] Shepperd S, Iliffe S, Doll HA (2016). Admission avoidance hospital at home. Cochrane Database Syst Rev.

[20] Caplan GA, Sulaiman NS, Mangin DA (2012). A meta-analysis of “hospital in the home.”. Med J Aust.

[21] Levine DM, Ouchi K, Blanchfield B (2020). Hospital-level care at home for acutely ill adults a randomized controlled trial. Ann Intern Med.

[22] Levine DM, Ouchi K, Blanchfield B (2018). Hospital-level care at home for acutely ill adults: a pilot randomized controlled trial. J Gen Intern Med.

[23] Watchorn R, Meys R, Jolliffe V (2014). Cold panniculitis in a horse rider. BMJ.

[24] Sonis JD, Berlyand Y, Yun BJ (2020). Patient experiences with transfer for community hospital inpatient admission from an academic emergency department. J Patient Exp.

[25] Berlyand Y, Thompson R, Yun BJ (2022). ED admin & clinical operations bringing hospital care into the home. SAEM Pulse.

[26] Alishahi Tabriz A, Birken SA, Shea CM (2019). What is full capacity protocol, and how is it implemented successfully?. Implement Sci.

[27] Yakobi R (2017). Impact of urgent care centers on emergency department visits. Health Care: Current Reviews.

[28] Kelen GD, Wolfe R, D’onofrio G (2021). Emergency department crowding: the canary in the health care system. NEJM Catalyst.

[29] Weiss SJ, Derlet R, Arndahl J (2004). Estimating the degree of emergency department overcrowding in academic medical centers: results of the National ED Overcrowding Study (NEDOCS). Acad Emerg Med.

[30] Weiss SJ, Ernst AA, Nick TG (2006). Comparison of the National Emergency Department Overcrowding Scale and the Emergency Department Work Index for quantifying emergency department crowding. Acad Emerg Med.

[31] Asplin BR, Magid DJ, Rhodes KV (2003). A conceptual model of emergency department crowding. Ann Emerg Med.

[32] McCarthy ML, Aronsky D, Jones ID (2008). The emergency department occupancy rate: a simple measure of emergency department crowding?. Ann Emerg Med.

[33] Peltan ID, Bledsoe JR, Oniki TA (2019). Emergency department crowding is associated with delayed antibiotics for sepsis. Ann Emerg Med.

[34] R Core Team (2022). R: A language and environment for statistical computing.

[35] Jaeker JAB, Tucker AL (2016). Past the point of speeding up: the negative effects of workload saturation on efficiency and patient severity. Management Science.

[36] Pines JM, Iyer S, Disbot M (2008). The effect of emergency department crowding on patient satisfaction for admitted patients. Acad Emerg Med.

