# Supplementary material for: Impact of Emergency Department Crowding on Discharged Patient Experience
Source: West J Emerg Med. 2022 Dec 31;24(2):185–92. doi: 10.5811/westjem.2022.10.58045 (PMC10047741; doi:10.5811/westjem.2022.10.58045)
Supplement: Supplementary file 1 [file wjem-24-185-s001.docx]

**Supplement to Impact of Emergency Department Crowding on Discharged Patient Experience**

This supplement contains several additional statistical analyses which support the main text. We include the following:

- Supporting Table 1: Four additional logistic regression models which estimate detractor likelihood distinguishing between overall patients in a care area and the split between ED boarders (awaiting an inpatient bed) and non-boarders in that area. (Both "adjusted" and "unadjusted" models, in the language of the manuscript, are included for each.)
- Supporting Table 2: A pairwise area under the curve (AUC) comparison of the three detractor likelihood models.
- Supporting Table 3: A logistic regression model that predicts the likelihood of a patient leaving against medical advice or without being seen.

­

**Supporting Table 1: Additional logistic regression models for estimating a patient’s detractor likelihood**

In contrast to the main text, these models distinguish between boarders versus non-boarders in the patient's care area. Boarder and non-boarders are standardized as before. *P* values below 0.05 are bolded. Odds ratios greater than 1.0 correspond to increased likelihood of being a detractor. Care Area A has zero boarders (for all index patients in this area) during the study period and therefore a coefficient is not estimated.

SE=standard error; CI=confidence interval; AUC=area under the curve; AMA=against medical advice; LWBS=left without being seen; NA=not applicable

**Supporting Table 2: Pairwise comparison of area under the curve for detractor likelihood models**

This table contains pairwise comparisons for equality of area under the curve (AUC) across the six detractor likelihood models (two from the main text Table 1 and four from Supporting Table 1), using DeLong's (two-sided) test for paired receiver operating characteristic (ROC) curves. Given the multiple comparisons, we apply a Holm correction to the *p* values shown in this table. Corrected *p* values below a 0.05 threshold are bolded.

**Supporting Table 3: Logistic regression model estimating likelihood of leaving against medical advice or without being seen**

The adjusted model contains the same patient- and encounter-specific covariates as the detractor likelihood models, except for length of stay (which is omitted given that it is effectively censored for patients leaving AMA or LWBS, the dependent variable of interest).

 SE=standard error; CI=confidence interval; AUC=area under the curve; AMA=against medical advice; LWBS=left without being seen; NA=not applicable
